# Comparison between sEMG and force as control interfaces to support planar arm movements in adults with Duchenne: a feasibility study

**DOI:** 10.1186/s12984-017-0282-6

**Published:** 2017-07-12

**Authors:** Joan Lobo-Prat, Kostas Nizamis, Mariska M.H.P. Janssen, Arvid Q.L. Keemink, Peter H. Veltink, Bart F.J.M. Koopman, Arno H.A. Stienen

**Affiliations:** 1 Department of Biomechanical Engineering, University of Twente, Drienerlolaan 5, Enschede, 7522 NB The Netherlands; 2 Department of Rehabilitation, Radboud University Medical Center, Reinier Postlaan 4, Nijmegen, 6500 HB The Netherlands; 3 Department of Biomedical Signals and Systems, University of Twente, Drienerlolaan 5, Enschede, 7500 AE The Netherlands; 4 Department of Physical Therapy and Human Movement Sciences, Northwestern University, 645 N Michigan Ave Suite 1100, Chicago (IL), 60611 USA

**Keywords:** Duchenne, Arm support, sEMG control, Force control, Stiffness compensation, Control interface, Assistive device

## Abstract

**Background:**

Adults with Duchenne muscular dystrophy (DMD) can benefit from devices that actively support their arm function. A critical component of such devices is the control interface as it is responsible for the human-machine interaction. Our previous work indicated that surface electromyography (sEMG) and force-based control with active gravity and joint-stiffness compensation were feasible solutions for the support of elbow movements (one degree of freedom). In this paper, we extend the evaluation of sEMG- and force-based control interfaces to simultaneous and proportional control of planar arm movements (two degrees of freedom).

**Methods:**

Three men with DMD (18–23 years-old) with different levels of arm function (i.e. Brooke scores of 4, 5 and 6) performed a series of line-tracing tasks over a tabletop surface using an experimental active arm support. The arm movements were controlled using three control methods: sEMG-based control, force-based control with stiffness compensation (FSC), and force-based control with no compensation (FNC). The movement performance was evaluated in terms of percentage of task completion, tracing error, smoothness and speed.

**Results:**

For subject S1 (Brooke 4) FNC was the preferred method and performed better than FSC and sEMG. FNC was not usable for subject S2 (Brooke 5) and S3 (Brooke 6). Subject S2 presented significantly lower movement speed with sEMG than with FSC, yet he preferred sEMG since FSC was perceived to be too fatiguing. Subject S3 could not successfully use neither of the two force-based control methods, while with sEMG he could reach almost his entire workspace.

**Conclusions:**

Movement performance and subjective preference of the three control methods differed with the level of arm function of the participants. Our results indicate that all three control methods have to be considered in real applications, as they present complementary advantages and disadvantages. The fact that the two weaker subjects (S2 and S3) experienced the force-based control interfaces as fatiguing suggests that sEMG-based control interfaces could be a better solution for adults with DMD. Yet force-based control interfaces can be a better alternative for those cases in which voluntary forces are higher than the stiffness forces of the arms.

## Background

Duchenne muscular dystrophy (DMD) is an X chromosome-linked recessive neuromuscular disease, which affects mainly males. It is diagnosed in childhood with an incidence of 1:5000 male live births [1]. Defective mutations in the dystrophin gene result in progressive degeneration of skeletal, respiratory and cardiac muscles. Generally people with DMD lose independent ambulation by the age of 12, followed by the development of scoliosis and deterioration of the upper extremity function during their teens, and develop severe cardiomyopathies and respiratory problems during their twenties [2, 3]. Over the last five decades, the lifespan of men with DMD has increased from 20 to 35 years due to improvements in health care and the introduction of home care technology, such as artificial ventilators [4]. As a result, there is currently a considerable group of adults with DMD living with severe physical impairments and a strong dependency on care [5].

People with DMD can benefit from commercially available passive arm supports that compensate for the weight of their arms [6, 7]. By the time they reach their twenties, the decrease of muscle force combined with the increase of passive joint-stiffness [8, 9] generally makes gravity compensation insufficient to support their arm function [10]. At that point, adults with DMD may benefit more from active arm supports, which can provide extra support and augment their residual capabilities. Active arm supports could enable them to continue performing basic activities of daily living and maintain social participation.

To control such devices, the user needs a way to communicate his motion intention to the device through a control interface [11]. Currently, the only control interfaces available for adults with DMD are hand joysticks and switches, which are used to control wheelchairs and external robotic arms. We consider that the use of control interfaces that detect the motion intention from physiological signals that are implicitly related to the supported motion can result in a more natural and intuitive interaction with the robotic arm support. Surface electromyography (sEMG) and force-based interfaces are two promising strategies for the control of active arm supports as they have been widely implemented in prostheses and orthoses/exoskeletons [11, 12].

The clinical standard sEMG-based control strategy implemented in upper limb prosthetics is a simple amplitude-based dual site control approach, also known as direct control [13]. This method measures sEMG from two independent residual muscles, or by distinguishing different activation levels of one residual muscle. Switching techniques such as muscle co-contraction are commonly implemented for enabling the sequential operation of different degrees of freedom (DOF). Direct control has also been implemented in upper-extremity orthoses [14, 15]. More advanced sEMG-based control strategies for operating active orthoses/exoskeletons are based on estimating joint angles or torques from the sEMG signals of the muscles that mainly contribute to the supported motion. Common estimation methods include pattern-recognition-based algorithms and regression-based algorithms [16]. sEMG-based control interfaces have been previously proposed for people with muscular weakness. Vogel et al. [17] evaluated the feasibility of using a sEMG regression-based algorithm (i.e. neural network) for the control of an external robotic arm in patients with spinal muscular atrophy. Another example is the study of Polygerinos et al. [18] that developed a sEMG controlled soft robotic glove for people with muscle dystrophy.

Force-based control interfaces can provide assistance by actively reducing the impedance of the user or the effect of external forces such as gravity forces [19]. These interfaces generally implement control strategies where the output motion is proportional to the input force (i.e. admittance control). Force-based control interfaces have been proposed in previous studies to support the arm function of people with muscular weakness. The study by Rahman et al. [10] evaluated a force-based control interface using a commercial SACRA robot with two healthy subjects (clinicians) and latter implemented a force-based control interface in the active version of the WREX exoskeleton that is under development [20]. In the ESTA project a force-controlled arm support was also under development for people with muscular weakness [21]. Corrigan and Foulds [22] investigated the implementation of admittance control for people with DMD using an external robotic arm. Despite these few examples, force-based controlled interfaces are mostly implemented in rehabilitation robots for patients that need training to regain motor control, mobility and strength [12, 23].

In our previous work [24] we investigated the feasibility of using sEMG, and force-based control with active gravity and joint-stiffness compensation. Three adults with DMD (Brooke score 5) performed a series of discrete position-tracking tasks using a one degree-of-freedom (DOF) active elbow support. Despite all three participants had not performed any voluntary movements with their arms for the 3–5 years prior to the study, all of them were 100% successful in completing the series of discrete position-tracking tasks with a reasonable average completion time using both control interfaces. Interestingly, sEMG based-control was perceived as less fatiguing by all three subjects. We presume this difference in fatigue is due to the fact that sEMG signals are not disturbed by gravity or joint-stiffness, and therefore can better produce the intended movement of the user compared to force signals. In conclusion, our previous results indicated that despite some performance differences both methods were feasible for the control of one-DOF active elbow supports by adults with DMD who have very limited arm function.

This paper extends the feasibility study on the use of sEMG and force as control interfaces from one to two DOF^1^. The goals of this study were: (I) to investigate whether adults with DMD can use sEMG-based control, force-based control with stiffness compensation (FSC) and force-based control with no compensation (FNC) to perform planar arm movements; (II) to evaluate their movement performance during a line-tracing task; and (III) to examine users’ acceptance of the control methods. The motivation for adding the FNC method into the present study (which was not tested in our previous feasibility study [24]) is that the FNC method resembles the dynamics of generally used passive planar arm supports, and therefore, we can indirectly compare the performance of passive arm supports with active arm supports controlled with sEMG and FSC.

## Methods

The feasibility of using sEMG and force-based control interfaces for supporting planar movements in adults with DMD was evaluated with three participants and using a commercial robotic manipulator as experimental arm support during a series of line-tracing tasks.

### General Framework for sEMG- and Force-based admittance control

Force- and sEMG-based control interfaces can be used in combination with an admittance model (Eq. 1) that maps the estimated force of the user ($\hat {F}_{vol}$), to the intended motion of the arm support (*v*_*ref*_). Using this control method, the interface dynamics of the device can be modified by changing the virtual parameters of the admittance model, which is usually composed of mass (*M*_*vir*_), damping (*D*_*vir*_), and stiffness (*K*_*vir*_; not used in our application).

1 $$  H_{adm}(s) = \frac{v_{ref}(s)}{\hat{F}_{vol}(s)} = \frac{1}{M_{vir}s + D_{vir}}  $$

where *s* is the Laplace transform variable. In order to have a control interface that is highly responsive to the low amplitude signals of people with DMD, the admittance model should have *M*_*vir*_ and *B*_*vir*_ as low as possible (i.e. highest admittance possible) but still high enough for the control to be stable and comfortable.

**Fig. 1:**
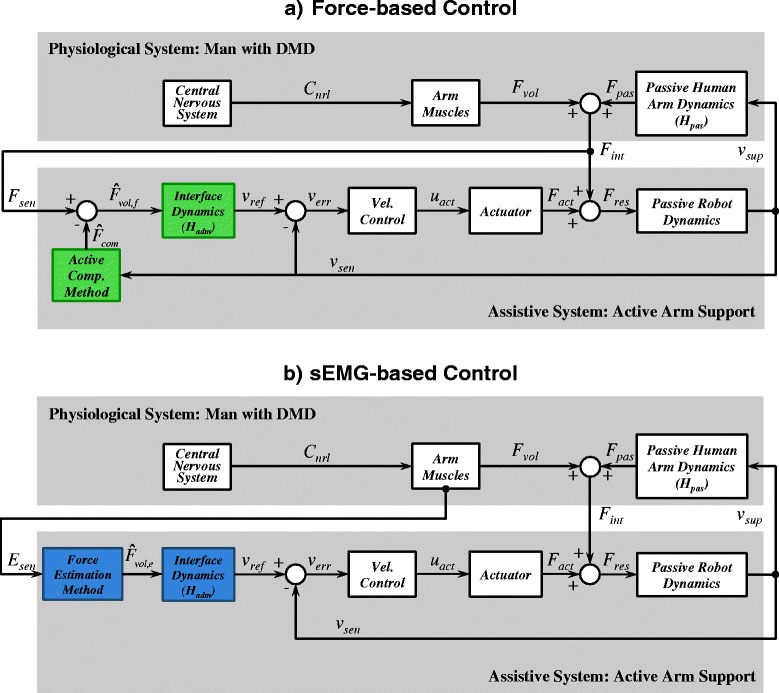
General Framework for sEMG- and Force-based Admittance Control. Simplified control diagram of the physiological system and the assistive system. To perform a movement the man with DMD generates neural commands (*C*_*cnt*_) with his central nervous system (CNS), which result in muscle activation (i.e. from where sEMG signals *E*_*sen*_ are measured) and muscle contraction that generates voluntary muscle force (*F*_*vol*_). Either force (*F*_*int*_) or sEMG signals (*E*_*sen*_) are used to derive the motion intention of the user and control the assistive system. **a** The interaction force (*F*_*int*_), which is a combination of the voluntary muscle force (*F*_*vol*_) and the passive/intrinsic human arm force (*F*_*pas*_) is measured by a force sensor (*F*_*int*_). An estimation of the voluntary force of the user is obtained by actively compensating the intrinsic arm force ($\hat {F}_{com}$). **b** sEMG signals from the arm muscles of the user are measured and a voluntary force is estimated from them. In both control methods the estimated voluntary force is used as input for an admittance model. The resulting velocity reference signal (*v*_*ref*_) is send to a low-level velocity feedback controller that operates the actuator. The resulting force (*F*_*res*_) generated by the actuator (*F*_*act*_) together with the interaction force (*F*_*int*_) moves the passive robot and human arm dynamics with a support velocity (*v*_*sup*_)

Figure 1 shows a simplified control diagram of sEMG- and force-based admittance control. To perform a movement the man with DMD generates neural commands (*C*_*nrl*_) with his central nervous system, which result in muscle activation (i.e. from where sEMG signals (*E*_*sen*_) are measured) and muscle contraction that generates voluntary muscle force (*F*_*vol*_). The muscle force, together with the force from the passive dynamics of the arm (*H*_*pas*_, Eq. 2), results in the interaction force between the device and the user (*F*_*int*_), which is measured by the force sensor.

2 $$  H_{pas}(s) = \frac{F_{pas}(s)}{v_{sup}(s)} = M_{pas}s + D_{pas} + \frac{K_{pas}}{s}.  $$

When using force-based control (Fig. 1 a) the force sensor measures the interaction force (*F*_*int*_), which contains not only the voluntary force of the user (*F*_*vol*_), but also the intrinsic (or passive) forces of the arm (*F*_*pas*_). Thus, to assist the movement intention of the user it is crucial to distinguish the voluntary force from the other components. In order to have an estimate of the voluntary force of the human ($\hat {F}_{vol,f}$), the “Active Compensation Method” subsystem in Fig. 1 a estimates the intrinsic arm forces ($\hat {F}_{com}$) using information from the arm movement (*v*_*sup*_). The estimated compensation force is then subtracted from the measured interaction force:

3 $$  \begin{aligned} &\hat{F}_{vol,f} = F_{int} - \hat{F}_{com}\text{, where }\\ &\hat{F}_{com} = \hat{F}_{ine}({v}_{sup}s) - \hat{F}_{dmp}({v}_{sup}) - \hat{F}_{stf}(v_{sup}/s), \end{aligned}  $$

the $\hat {}$ denotes that a value is estimated, $\hat {F}_{ine}$ indicates the estimated arm inertia force, $\hat {F}_{dmp}$ indicates the estimated arm damping force and $\hat {F}_{stf}$ indicates the estimated arm stiffness force. In the present study we limited the active compensation to arm stiffness forces since these are the dominant components in low-frequency arm movements, which are the ones we intend to support for the performance of activities of daily living (ADL). Moreover, it has been found that joint stiffness is significantly increased for people with DMD [8]. Nevertheless, note that adding active compensation of damping and inertia forces of the arm could provide extra assistance to the users [19, 25, 26].

In the case of sEMG-based control (Fig. 1 b), the voluntary force of the user ($\hat {F}_{vol,e}$) is directly estimated from the sEMG signals. Note that, in contrast to force-based control interfaces, the sEMG signals are not affected by the passive arm dynamics, and therefore, do not require any compensation method.

### Participants

Three adults with DMD participated in this study (18–23 years old). Participants were carefully selected considering that they should have diverse levels of arm function with Brooke scores [27] of 4, 5 and 6. We choose participants with diverse levels of arm function to explore the feasibility of the control interfaces in different stages of the disease. Demographic information of the subjects is shown in Table 1.

**Table 1:** Demographic and arm function information of the participants

Subject’s	Age	Brooke	Preferred	Maximum	Arm
Code	(years)	scoore	Arm	Force (N)	Function
1	22	4	Right	22	Can raise hands to mouth, but cannot raise 200 g to mouth
2	18	5	Right	6	Cannot raise hands to mouth, but can use hands to hold a pen or to pick up coins
3	23	6	Right	3	Cannot raise hands to mouth and has no useful function of the hands

### The Setup

**Fig. 2:**
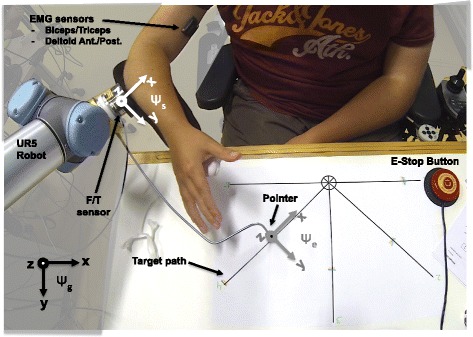
The research setup. Subject S1 controlling the active arm support during the evaluation of one of the control interfaces. The task of the subject was to trace the 5 target paths with the pointer that was connected to the endpoint of the UR5 Robotic Arm. The UR5 Robotic Arm was used as an active arm support which could be controlled with sEMG- and force-based control interfaces with no compensation (FNC) and with stiffness compensation (FSC)

**Fig. 3:**
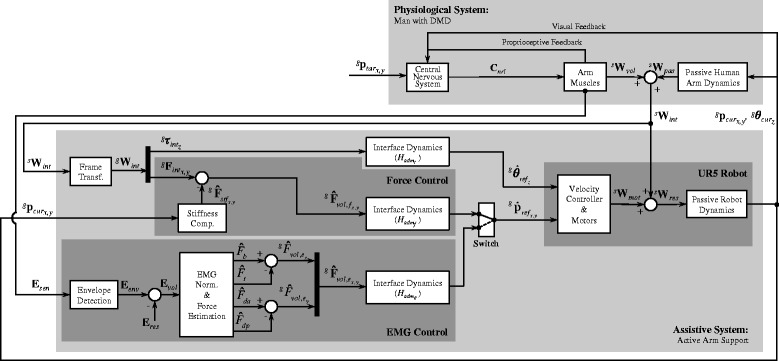
The control diagram implemented in the active arm support. The upper section represents the physiological system (man with DMD), while the lower section represents the assistive system (active arm support). To perform a movement and reach the target position ($p_{tar_{x,y}}$) the man with DMD generates neural commands (*C*_*cnt*_) with his central nervous system (CNS) that result in muscle activation (i.e. from where sEMG signals **E**_*sen*_ are measured), and in muscle contraction that generates a voluntary wrench (**W**_*vol*_). The intention of the user is detected in two ways: from sEMG signals (**E**_*sen*_) or by measuring the interaction force and torque between the user’s arm and the active arm support (**W**_*int*_). The interaction wrench (**W**_*int*_), which is a combination of the voluntary wrench (**W**_*vol*_) and the passive/intrinsic human arm wrench (**W**_*pas*_) is measured by a force/torque sensor (**W**_*int*_). In the force-based control method with stiffness compensation (FSC) an estimation of the voluntary force of the user ($\hat {\mathbf {F}}_{vol_{x,y}}$) is obtained by actively compensating the stiffness forces of the arm ($\hat {F}_{stf}$). The estimated stiffness forces for a given position of the arm ($\mathbf {p}_{pnt_{x,y}}$) are obtained from previously measured data. In the force-based control method without stiffness compensation (FNC) the estimated voluntary forces ($\hat {\mathbf {F}}_{vol_{x,y}}$) are equal to the measured interaction forces ($\mathbf {F}_{int_{x},y}$). In the sEMG-based control method the sEMG signals from two agonist/antagonist muscle pairs (biceps/triceps, and deltoid anterior/posterior) are measured and non-physiological voluntary forces are estimated from each muscle (*F*_*b*_,*F*_*t*_,*F*_*da*_,*F*_*dp*_). An estimated voluntary force in the *x* and *y* directions ($\hat {\mathbf {F}}_{vol_{x,y}}$) are obtained by subtracting the estimated voluntary forces of the antagonist muscles from the agonist muscles. In all control methods the estimated voluntary forces are used as input to an interface dynamic system ($H_{adm_{e}},H_{adm_{f}}$) that rendered the dynamics of a mass-damper system. The rotational velocity of the pointer around the vertical axis ($\dot {\theta }_{ref_{z}}$) is actively driven by the interaction torque between the subject’s arm and the robot ($\tau _{int_{z}}$) using the interface dynamics ($H_{adm_{t}}$). The resulting linear and angular velocity reference signals ($\dot {\mathbf {p}}_{ref_{x,y}},\dot {\theta }_{ref_{z}}$) are send to a low-level velocity controller of the UR5 Robotic Arm. The wrench (**W**_*res*_) generated by the motors of the UR5 Robotic Arm (**W**_*mot*_) together with the interaction wrench (**W**_*int*_) moves the passive robot dynamics together with the pointer and the human arm dynamics to the position $\mathbf {p}_{pnt_{x,y}}$ and the orientation $\theta _{pnt_{z}}$. This motion is measured by the proprioceptive sensors of the man with DMD and is used to generate new neural commands to eventually reach the target position

The UR5 Robotic Arm (UR5, Universal Robots, Denmark) was used as a research platform to provide active support for planar arm movements (Fig. 2). A plastic forearm cuff from the Darwing arm support (Focal Meditech, Tilburg, The Netherlands), with a custom-made wrist support was attached to the end-point of the robot. Between the arm cuff and the end-point of the robot, a 6-DOF force/torque sensor (ATI mini 45, Industrial Automation, USA) was mounted to measure the interaction force and torque between the user and the robot (^*s*^ **W**_*int*_ in Fig. 3). Four differential sEMG wireless electrodes (Trigno Lab, Delsys, USA) were used to measure sEMG signals (**E**_*sen*_ in Fig. 3) from four arm muscles: biceps, triceps, deltoid anterior and deltoid posterior. A metal rod with a marker attached at the end of it was mounted on the last segment of the UR5 Robotic Arm and served as a pointer for the tracing task.

Both the sEMG- and the force-based interfaces controlled the horizontal linear velocities of the pointer ($^{g}{\dot {\mathbf {p}}}_{{ref}_{x,y}}$) in the global reference frame (*ψ*_*g*_ in Fig. 2). In both control methods, the rotational velocity of the pointer around the vertical axis ($^{g}\dot {\theta }_{ref_{z}}$) was actively driven by the interaction torque between the subject’s arm and the robot. In this way, the task of the experimental subject was reduced to controlling the position of the pointer in the plane (i.e. two-DOF tasks), and the orientation of the arm was automatically given by the musculoskeletal constraints acting on the human arm. The UR5 Robotic Arm controller was programed to control the velocity of a virtual end-point that was set to coincide with the endpoint frame (*ψ*_*e*_ in Fig. 2). The remaining three DOF of the robot’s virtual endpoint were locked ($^{g}\dot {p}_{ref_{z}}=0;^{g}{\boldsymbol {\dot {\theta }}}_{ref_{x,y}}=[\!0,0]$)^2^.

The analog signals from the force/torque sensor and the sEMG signals were measured by a real-time computer (xPC Target 5.1, MathWorks Inc., USA) by means of a National Instruments card (PCI-6229; National Instruments Corp., USA), which performed the analog-to-digital conversion at a sampling frequency of 1 kHz and 16-bit resolution. The controller was also running on the real-time computer and was sending the velocity commands (using the URscript function *speedl* [28]) at 125 Hz through UDP/IP communication to a Windows PC, which at the same time was communicating with the UR5 Robotic Arm via TCP/IP communication at a frequency of 125 Hz. The Windows PC that interfaced between the robot controller and the real-time computer was required because TCP/IP communication was not supported by the real-time computer and UDP/IP communication was not supported by the UR5 Robotic Arm.

### Signal processing and control

The participants performed a line-tracing task with the goal of reaching the end point of the target line ($\mathbf {p}_{tar_{x,y}}$). The central nervous systems received proprioceptive and visual information about the current pointer position ($\mathbf {p}_{pnt_{x,y}}$) and the target position, and sent neural commands (**C**_*nrl*_) to the arm muscles in order to perform the task. The intention of the user was detected in two ways: (I) by measuring sEMG signals (**E**_*sen*_); or (II) by measuring the interaction force and torque, between the user’s arm and the active arm support (^*s*^ **W**_*int*_).

#### sEMG-based control

As in our previous study [24], the sEMG-based control interface implemented was based on the method known as direct or proportional control. In the present study we used the muscle activation of two agonist/antagonist pairs (biceps/triceps, and deltoid anterior/posterior) to obtain indirect and non-physiological force estimations that control Cartesian movements in global *x* and *y* directions (Fig. 2). Note that we measured sEMG signals from muscles that are not directly related to the supported movement, which can be considered as a non-intuitive mapping. Our reasoning behind this choice was to use sEMG signals from muscles that we knew from previous pilot trials in adults with DMD that could give a relatively good signal quality. Following this control strategy if the subject only activates the biceps the end point of the robotic arm moves in the positive *x* direction; if only the triceps is active the end point moves in the negative *x* direction; if only the deltoid anterior is active the end point moves in the positive *y* direction; and if only the deltoid posterior is active the endpoint moves in the negative *y* direction. Diagonal movements are then performed by simultaneously activating the biceps or the triceps and the deltoid anterior or posterior.

The processing of the sEMG signals’ envelopes (${E}_{env_{k}}$) was performed by using a full-wave rectification and filtering with a 2^*nd*^ order Butterworth filter with a cut-off frequency of 3 Hz. The filter settings were chosen in line with previous studies on sEMG control [14, 15, 24, 29] and pilot trials on our setup to find a fair tradeoff between signal bandwidth and phase-lag. The voluntary sEMG signals (${E}_{vol_{k}}$) and the estimated muscle forces ($\hat {F_{k}}$) were derived using:

4 $$ \hat{F_{k}} = \alpha \frac{E_{vol_{k}}}{E_{mvic_{k}}}\text{, where }E_{vol_{k}}= E_{env_{k}} - E_{res_{k}},  $$

subscript *k* represents the abbreviations of the biceps (*b*), triceps (*t*), deltoid anterior (*da*) and deltoid posterior (*dp*) muscles, $E_{env_{k}}$ denotes the processed sEMG envelope signal, $E_{res_{k}}$ represents the average of the processed sEMG envelope signal during rest, and $E_{mvic_{k}}$ represents the mean maximum magnitude of $E_{env_{k}}$ over three seconds of maximum voluntary isometric contraction (MVIC). Note that for consistency of the units *α*=1 and has units of newtons to obtain $\hat {F_{k}}$ in newtons. Finally, the estimated voluntary force $^{g}\hat {F}_{vol,e}$ for each controlled DOF of the endpoint (i.e. x and y directions) was obtained by subtracting the estimated antagonist muscle force from the estimated agonist muscle force:

5 $$ ^{g}\hat{F}_{vol,e_{x}} = \hat{F}_{b} - \hat{F}_{t} \text{and }^{g}\hat{F}_{vol,e_{y}} = \hat{F}_{da} - \hat{F}_{dp},  $$

where the superscript *g* denotes that the estimated force is expressed in the global coordinate frame (*ψ*_*g*_ in Fig. 2).

#### Force-based control

**Fig. 4:**
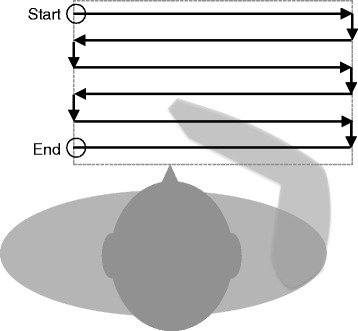
Measurement path to obtain a two-dimensional stiffness force field. Graphical representation of the path made by the robot across the participant’s workspace (*dashed square*) and used to obtain a two-dimensional stiffness force field. This measurement data is used to actively compensate arm stiffness forces when using the force-based control with stiffness compensation (FSC) method

For the force-based control interface, the interaction force and torque, denoted with a wrench (^*s*^ **W**_*int*_), were measured with the force/torque sensor $\left ({\!~\!}^{s}\mathbf {W}_{int}=\left [{\!~\!}{\!~\!}^{s}{\boldsymbol {\tau }}_{int}{\!~\!}^{T},^{s}\mathbf {F}_{int}{\!~\!}^{T}\right ]^{T}\right)$ and expressed in the sensor frame (*ψ*_*s*_). The measured wrench was transformed to the endpoint coordinate system (*ψ*_*e*_) using standard rotation and transformation matrices and rotated to match the orientation of the global coordinate system (*ψ*_*g*_). With the measured wrench expressed in the global frame (^*g*^ **W**_*int*_), the estimated stiffness compensation force $\left ({\!~\!}^{g}\hat {\mathbf {F}}_{stf_{x,y}}({\!~\!}^{g}\mathbf {p}_{cur_{x,y}})\right)$ was subtracted from the horizontal measured force $\left ({\!~\!}^{g}\mathbf {F}_{int_{x,y}}\right)$ to obtain an estimate of the voluntary force applied by the user $\left ({\!~\!}^{g}\hat {\mathbf {F}}_{vol,f_{x,y}}\right)$. The estimated stiffness compensation force was obtained by performing a force measurement during a slow movement of the arm along a predefined grid that covered the participant’s workspace (see Fig. 4). The arm of the participant was moved by the robot in position control mode while the user was relaxed. After this measurement a two-dimensional linear interpolation was applied (using the Matlab function *interp2*) to achieve a force field with a uniform resolution (i.e. 50×50 points).

The force field was then used for the force-based control with stiffness compensation (i.e. FSC) by subtracting the interpolated values of the force-field for a specific *x* and *y* position of the endpoint (${\!~\!}^{g}\mathbf {p}_{cur_{x,y}}$):

6 $$ {\!~\!}^{g}\hat{\mathbf{F}}_{vol,f_{x,y}} =\;{\!~\!}^{g}\mathbf{F}_{int_{x,y}} -\;^{g}\hat{\mathbf{F}}_{stf_{x,y}}\left({\!~\!}^{g}\mathbf{p}_{cur_{x,y}}\right).  $$

For the force-based control with no compensation (i.e. FNC), the force expressed in the global reference frame was equal to the estimated voluntary force $\left ({\!~\!}^{g}\mathbf {F}_{int_{x,y}} ={\!~\!}^{g}\hat {\mathbf {F}}_{vol,f_{x,y}}\right)$. A more detailed description of the stiffness force field interpolation method can be found in [30].

#### Admittance model

Depending on which control method was used, the estimated force resulting from the sEMG-based or the force-based control (i.e. $^{g}\hat {\mathbf {F}}_{vol,f_{x,y}}$ or $^{g}\hat {\mathbf {F}}_{vol,e_{x,y}}$) was mapped to linear velocities ($^{g}\dot {\mathbf {p}}_{ref_{x,y}}$) using an admittance model (i.e. $H_{adm_{e}}$ or $H_{adm_{f}}$) that rendered the dynamics of a mass-damper system. The torque signal around the vertical axis ($^{g}\tau _{sen_{z}}$) was also mapped to angular velocity ($^{g}\dot {\theta }_{ref_{z}}$) using an admittance model ($H_{adm_{t}}$) that rendered the dynamics of an inertia-damper system:

7 $$ H_{adm_{c}} = \frac{1}{M_{vir_{c}}s+D_{vir_{c}}}.  $$

where subscript *c* stands for the type of control input: torque (*t*), sEMG (*e*) or force (*f*). The vector of the linear reference velocities ($^{g}\dot {\mathbf {p}}_{ref_{x,y}}$) was combined with the rotational velocity reference ($^{g}\dot {\theta }_{ref_{z}}$) and sent to the UR5 Robotic Arm. The velocity controller of the UR5 Robotic Arm made the motors apply a wrench (^*s*^ **W**_*mot*_) that in combination with the interaction wrench (^*s*^ **W**_*int*_) moved the passive dynamics of the robot and of the human arm.

Taking into account that force and sEMG signals present differences due to their origin (EMG: muscle activation, force: muscle contraction), we ensured that after the signal processing both control inputs ($\hat {\mathbf {F}}_{vol,e}$ and $\hat {\mathbf {F}}_{vol,f}$) presented bandwidths higher than the one of the human movement controller during ADL (i.e. around 2 Hz [10, 31]).

Regarding the choice of the interface dynamics, although it may seem logical to use the same parameters to compare the control methods, we found that the intrinsic differences between them do not make this possible. First, there is a difference in units. Despite that both force and sEMG signals are measured in volts, only force signals are properly scaled to newtons. The sEMG signals have no force equivalent unit to scale them to. Therefore, actual admittance control (force as input and velocity/position as output) is only possible using force signals, and any kind of admittance control using estimated forces from sEMG signals (or other signals) as input will be pseudo-admittance control. Second, the sEMG-based control system proposed in this study is based on obtaining indirect and non-physiological force estimations from the activation difference between agonist and antagonist muscle pairs to assist Cartesian movements in the horizontal plane. Therefore, these voluntary force estimations generated from the sEMG-based control method are not directly comparable to the voluntary force estimations generated by the force-based control method. In other words, setting the parameters of the interface dynamics to the same values for both control methods does not imply a fair comparison.

Taking into account these differences between sEMG and force signals, we found that the best way to perform this comparative study was by defining the control methods as the combination of (1) a specific signal acquisition method, (2) a specific signal processing method and (3) specific interface dynamics (i.e. admittance parameters) together. The parameters of the interface dynamics for both control interfaces (Table 2) were then chosen from several pilot trials in order to obtain a similar responsiveness, comfort and stable interaction between the robot and the user. In the case of force-based control it was crucial to select interface dynamics that rendered a high admittance in order to provide assistance to the intended movement. Therefore, after performing the pilot trials we chose a damping and mass values ($M_{vir_{f}} = 15$ kg, $D_{vir_{f}} = 5$ Ns/m) that were as low as possible taking into account that the interaction between the robotic manipulator (i.e. UR5 Robotic Arm) and the human arm had to remain always safe and stable. When we tried to use the same parameters for the sEMG-based control method, the pilot subjects felt that movements were too fast and did not feel comfortable. This change was most likely due to the stabilizing effect of the passive human arm dynamics, an effect that is only present in the force-based control (i.e. the measured force captures the closed loop interaction between human arm and assistive device) and not in the sEMG-based control. In an effort to optimize the interface dynamics of each control method, we decided to increase the damping ($D_{vir_{e}} = 10$ Ns/m) in order to reduce the speed of the movements and make the participants feel comfortable again, and lower the mass ($M_{vir_{e}} = 10$ kg) in order to have a system that would still be sensitive enough.

**Table 2:** Virtual mass and damping parameters of the interface dynamics

	$M_{vir_{c}}$	$D_{vir_{c}}$
$H_{adm_{e}}$	10kg	10Ns/m
$H_{adm_{f}}$	15kg	5Ns/m
$H_{adm_{t}}$	2kgm^2^	1Nms/rad

Subscript *c* stands for the type of control input: torque (*t*), sEMG (*e*) or force (*f*)

### Experimental protocol

**Fig. 5:**
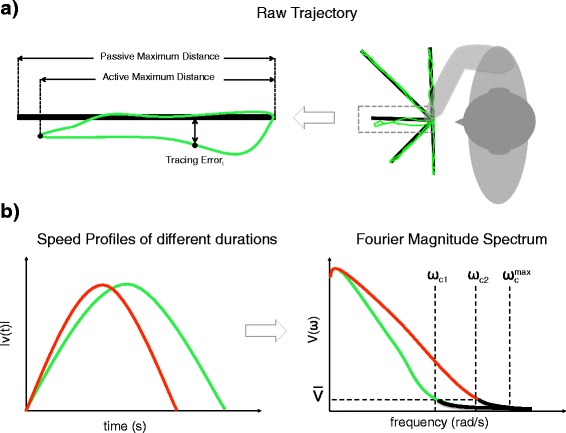
Graphical explanation of the performance metrics. **a** Illustration of the raw movement trajectory (*green*) and the distances used to calculate the percentage of task completion (PTC) the tracing error (TE). **b** Illustration of the spectral arc length (SPARC) derived from the Fourier magnitude spectrum of the speed profile. The green and red curves illustrate the speed profiles in time (*left*) and frequency (*right*) domain of two movements with different duration. The SPARC measure calculates the spectral arc length with a magnitude threshold ($\overline {V}$) that selects a cut-off frequency (*ω*_*c*_) for each power spectrum signal, making the smoothness measure independent of the movement duration

**Fig. 6:**
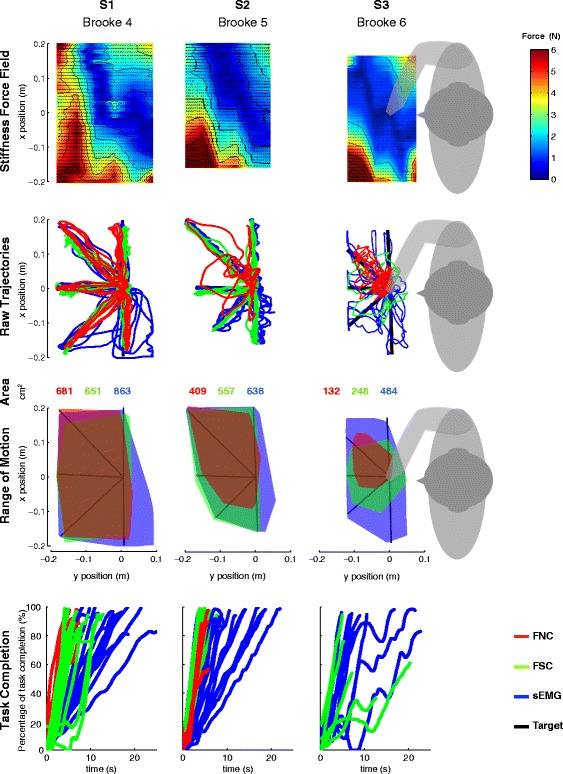
Stiffness force-fields, raw trajectories, reachable ROM and percentage of task completion over time. Data presented for each subject (*columns*) and each metric (*rows*). Stiffness force fields present similarities over participants with maximum stiffness forces of 6 N at the upper-left corner of the participant’s workspace and a region of low stiffness force going from the center of their chest to the right upper side of their workspace. Smoothness of the trajectories, reachable ROM and percentage of task completion rate differed with the level of arm function of the participants. Note that the FNC control method was not usable for subject S3 (Brooke 6)

The participants were invited to the Rehabilitation Department of the Radboud University Medical Center. After a detailed explanation of the purpose and procedure of the experiment, the participants placed their wheelchair in front of a height-adjustable table that was used to present the tracing task to the subjects. A comfortable position of the arm cuff and the amount of padding was adjusted to the convenience of the participants. Afterwards, the sEMG electrodes were placed and the sEMG signals were checked. The MVIC for each muscle was measured with the arm attached in the arm cuff and instructing the participant to perform three seconds of MVIC for each of the four targeted muscles. The participant had visual feedback of the raw sEMG signals and the envelopes of the sEMG signals during the measurement of the MVIC. Subsequently, the passive range of motion (ROM; shown as black lines in Figs. 5 and 6) of the participants was defined using the active arm support in force-based admittance control with a high virtual damping (i.e. $D_{vir_{f}}=\text {}20\text {Ns/m}$) while the researcher moved the arms of the participants along the target lines. Once the MVIC of the sEMG signals and the passive ROM were measured, the training for the sEMG-based control started.

#### sEMG-based Control:

First, movements along the *x* direction were trained for a maximum of 15 min (only usign biceps/triceps activations to generate $^{g}\hat {F}_{vol,e_{x}}$). Subsequently, movements in the *y* direction were trained for the same amount of time (only using deltoind anterior/posterior activations to generate $^{g}\hat {F}_{vol,e_{y}}$), and finally movements in both *x* and *y* directions simultaneously were trained for 15 min while practicing the line-tracing task. The line-tracing task consisted of tracing the five target lines (see black lines in Fig. 2) as accurate and fast as possible in a clock-wise direction, i.e. starting from the line pointing towards the subject’s left side and finishing with the line pointing to the subject’s right side. After the training phase, the subject performed the evaluation trials, which consisted of tracing the five lines three times (i.e. 3 repetitions, 1 repetition = 5 movements). Once the evaluation trials with the sEMG-based control interface were finished, the participant had a break of 45 to 60 min.

#### Force-based control:

After the break, the FSC and FNC methods were evaluated. First, the participant was relocated to the same position in front of the height-adjustable table. Subsequently, the subject trained with FNC for 15 min while practicing the line-tracing task. Once the training phase was completed, the subject performed the evaluation trials. After completing the evaluation trials with FNC, the subject relaxed for approximately 5 min while the measurement of the stiffness force field was performed. After the measurement of the stiffness force field the subject trained for 15 min with the FSC while practicing the line-tracing task. Once the training phase was complete, the subject performed the evaluation trials.

#### Questionnaire:

To evaluate the experience of the participants and the acceptance of the sEMG, FNC and FSC control interfaces, each participant was asked to answer 7 questions (see Table 3) after completing the trials with all control methods.

**Table 3:** Questions and preferences of the participants

	sEMG	FNC	FSC
Which interface…	Preference	Preference	Preference
1 …could control the arm support most accurate?	greenS2	redS1	blueS3
2 …could control the arm support fastest?	greenS2 blueS3		redS1
3 …did react best to your intention?	greenS2	redS1	blueS3
4 …was least fatiguing to use?	greenS2 blueS3	redS1	
5 …was the most easy to set up/install?		redS1 greenS2 blueS3	
6 …was the most comfortable to use?	greenS2 blueS3	redS1	
7 …has your overall preference?	greenS2 blueS3	redS1	

The codes of the subjects are color-coded

### Data analysis

Data analysis was performed on metrics derived from the end-point trajectories as function of time while tracing the five target lines. The movement performance was evaluated in terms of percentage of task completion (PTC), tracing error (TE), smoothness (SM) and speed (SP; see Table 4 and Fig. 5 for definitions). The chosen performance descriptors are common measures used in studies that evaluate the performance of control interfaces [32–34]. For every metric, its mean value through all the trials per interface and per subject was calculated together with the median and the standard deviation. The percentage of task completion and the tracing error were calculated using:

8 $$\begin{array}{*{20}l} PTC&=\frac{\text{Active Maximum Distance}}{\text{Passive Maximum Distance}}\cdot100, \end{array} $$

**Table 4:** Performance metrics

Performance Metric	Description
Percentage of Task Completion (%):	Ratio between the maximum active distance and the maximum passive distance for each of the five directions expressed as a percentage.
Tracing Error (cm):	Mean distance between each position of the endpoint’s trajectory and the target line.
Smoothness (-):	Spectral arc length (SPARC). The length of the frequency spectrum of the speed profile of a movement. [35].
Speed (cm/s):	The mean speed of a movement.

9 $$\begin{array}{*{20}l} TE&=\frac{\sum\limits_{i=1}^{n} \text{Tracing Error}_{i}}{n}. \end{array} $$

The smoothness metric was adopted from Balasubramanian et al. [35]. The authors developed a smoothness metric based on the spectral arc length (SPARC) of the speed profile. We calculated the SPARC for the Euclidean norm of the movement velocity using:

10 $$ \begin{aligned} SPARC&\triangleq-{\int_{0}^{\omega_{c}} \sqrt{\left(\frac{1}{\omega_{c}}\right)^{2}+\left(\frac{d\hat{V}(\omega)}{d\omega}\right)} d\omega},\>\>\>\>\>
\hat{V}(\omega)\triangleq\frac{V(\omega)}{V(0)}, \end{aligned}  $$

11 $$\begin{array}{*{20}l} \omega_{c}&\triangleq \text{min}\left\{ {\omega_{c}^{max}}, \underset{\omega}{\min}\left\{{\omega} \vert \hat{V}(r)\ <\overline{V} \ \forall \ r>\omega \right\}\right\}, \end{array} $$

where *V*(*ω*) is the Fourier magnitude spectrum of the speed profile (*v*(*t*)), and *ω*_*c*_ is the frequency bandwidth for which the SPARC is measured. The *ω*_*c*_ is adaptively selected based on a chosen threshold $\overline {V}$ and it is limited to a chosen maximum frequency ($\omega _{c}^{max}$). This makes possible the comparison of movements with different duration (e.g. green and red curves in Fig. 5 b) [35]. The parameters chosen in our analysis where $\overline {V}$=0.01 and $\omega _{c}^{max}$= 20 *π*. The value for $\omega _{c}^{max}$ was chosen following the recommendations in [35], and the value of the threshold ($\overline {V}$) was chosen after observing the magnitude spectrum of the velocity. By definition the SPARC metric has a negative value and values closer to zero indicate smoother motion.

A detailed inspection of the distribution of all metrics by control system for each subject was performed using box plots. The data points above or below 1.5 times the inter-quartile range are shown as outliers with a ‘+’ symbol. The statistical analysis was performed using the non-parametric Friedman’s tests (significance level of *p*<0.05) together with a post hoc analysis using the Wilcoxon rank-sum tests with a Bonferroni correction (resulting in a significance level of *p*<0.0167). The statistical tests were performed with R software (R Development Core Team 2015).

A representation of the trajectories was done by illustrating the raw trajectories, the reachable ROM, and the percentage of task completion as function of time for each subject and control interface. The representation of the reachable ROM was done for each control method by finding the smallest convex polygons that contained all the data points of the movement trajectories. The *convhull* function from Matlab was used to create the polygons.

## Results

Figure 6 illustrates the stiffness force fields, the raw endpoint trajectories, the reachable ROMs and the percentage of task completion over time during the line-tracing tasks using the sEMG, FNC and FSC control methods. For all subjects, the endpoint stiffness forces presented a maximum magnitude of 6 N at the upper-left corner of the participant’s workspace. In all subjects we also found a region of low endpoint stiffness force going from the center of their chest to the right upper side of their workspace.

**Fig. 7:**
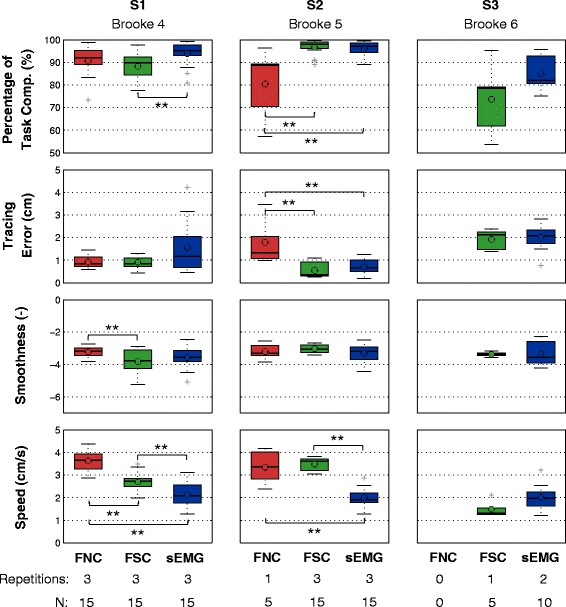
Box Plots and Wilcoxon Rank-Sum Tests of the performance metrics. Data presented for each subject (columns) and each performance metric (rows). The number of repetitions performed by the subjects with each method, and the number of observations (N) of each boxplot is indicated at the bottom of the figure. Subject S1 (Brooke 4) showed a high overall performance completing all three repetitions with all three control methods, and presents the best movement performance when using the FNC method. The low number of repetitions, the low percentage of task completion, and the high tracing error of FNC clearly indicated that subject S2 (Brooke 5) performed better with FSC and sEMG than with FNC. Movements of subject S2 presented significantly lower speed with sEMG than with FSC. Subject S3 (Brooke 6) was not able to effectively use neither of the force-based control interfaces (i.e. no movements completed with FNC and one repetition completed with FSC) and showed a better performance in terms of percentage of task completion and speed with sEMG and could reach almost his entire workspace. (**) indicates *p*<0.0167

Table 5 and Fig. 7 summarize the statistical analysis done on each performance metric. Significant differences were found among control methods according to Friedman’s test in terms of percentage of task completion and speed in the case of subjects S1 and S2, as well as in terms of tracing error for subject S2. Control methods did not significantly differ in terms of smoothness for any subject, albeit the p-value was much lower (*p*=0.057) in the case of subject S1, in which the Wilcoxon test detected a significant difference between FNC and FSC. None of the metrics indicated significant differences among control methods for subject S3. We present a detailed description of the results by subject in the rest of this section.

**Table 5:** Summary of the means and standard deviations and statistical tests for each performance metric, interface and subject

Metric			S1	S2	S3
PTC (%)	Interface	FNC	90.5(±6.5)	80.3(±14.9)	-
		FSC	88.2(±5.6)	96.5(±3.4)	73.6(±15.3)
		sEMG	93.9(±5.3)	95.9(±3.2)	85(±7.1)
	Friedman’s Test		***p*** **=** **0** **.** **0** **1** **2**	***p*** **=** **0** **.** **0** **2** **2**	*p*=0.179
		FNC-FSC	*p*=0.366	***p*** **=** **0** **.** **0** **0** **2**	-
	Wilcoxon Test	FSC-sEMG	***p*** **=** **0** **.** **0** **0** **4**	*p*=0.389	*p*=0.099
		sEMG-FNC	*p*=0.089	***p*** **=** **0** **.** **0** **0** **5**	-
		FNC	0.92(±0.26)	1.78(±1.04)	-
TE (cm)	Interface	FSC	0.91(±0.25)	0.54(±0.34)	1.92(±0.46)
		sEMG	1.56(±1.13)	0.71(±0.35)	2.01(±0.58)
	Friedman’s Test		*p*=0.420	***p*** **=** **0** **.** **0** **1** **5**	*p*=0.655
		FNC-FSC	*p*=0.967	***p*** **=** **0** **.** **0** **0** **2**	-
	Wilcoxon Test	FSC-sEMG	*p*=0.249	*p*=0.115	*p*=0.953
		sEMG-FNC	*p*=0.216	***p*** **=** **0** **.** **0** **0** **7**	-
		FNC	−3.2(±0.34)	−3.19(±0.47)	-
SM (-)	Interface	FSC	−3.83(±0.75)	−3.02(±0.26)	−3.37(±0.15)
		sEMG	−3.5(±0.68)	−3.29(±0.52)	−3.35(±0.69)
	Friedman’s Test		*p*=0.057	*p*=0.549	*p*=0.179
		FNC-FSC	***p*** **=** **0** **.** **0** **1** **6**	*p*=0.394	-
	Wilcoxon Test	FSC-sEMG	*p*=0.486	*p*=0.148	*p*=0.594
		sEMG-FNC	*p*=0.148	*p*=0.932	-
		FNC	3.27(±0.39)	3.35(±0.7)	-
SP (cm/s)	Interface	FSC	2.70(±0.38)	3.48(±0.2)	1.49(±0.35)
		sEMG	2.13(±0.53)	2(±0.3)	2.02(±0.57)
	Friedman’s Test		***p*** **<** **0** **.** **0** **0** **1**	***p*** **=** **0** **.** **0** **2** **2**	*p*=0.655
		FNC-FSC	***p*** **<** **0** **.** **0** **0** **1**	*p*=0.930	-
	Wilcoxon Test	FSC-sEMG	***p*** **=** **0** **.** **0** **0** **2**	***p*** **<** **0** **.** **0** **0** **1**	*p*=0.129
		sEMG-FNC	***p*** **<** **0** **.** **0** **0** **1**	***p*** **<** **0** **.** **0** **0** **1**	-

Bold *p*−*v* *a* *l* *u* *e* *s* indicate a signinfcant difference

### Subject S1 - Brooke score: 4

Subject S1 performed the complete number of repetitions with all three methods (three repetitions with each method resulting in N=15). The trajectories of subject S1 showed a high percentage of task completion with all control methods (FNC: 90.5±6.5%, FSC: 88.2±5.6%, sEMG: 93.9±5.3%; Fig. 7, Table 5). Trajectories with sEMG presented the highest mean percentage of task completion, with a significant difference (*p*=0.004) compared to FSC. No significant differences were found in terms of tracing error between the control methods. The trajectories using FNC show the highest smoothness (−3.2±0.3) with a significant difference compared to FSC (−3.8±0.8;*p*=0.0164). The mean speed of the trajectories presented significant differences (*p*<0.005) between all control methods: sEMG was the slowest (2.1±0.5 cm/s), FNC was the fastest (3.3±0.4 cm/s) and FSC was in between the other two (2.7±0.4 cm/s). The results of the questionnaire showed a clear preference for FNC with the exception of speed, for which subject S1 indicated that he could move the fastest with FSC.

### Subject S2 - Brooke score: 5

Subject S2 performed only one repetition with the FNC method (N = 5) and three repetitions with the other two methods (N=15). Subject S2 showed a significantly (*p*<0.01) worse performance in terms of percentage of task completion and tracing error when using FNC compared to FSC and sEMG-based control. While trajectories with FSC and sEMG showed a percentage of task completion close to 100% (FSC: 96.5±3.4%, sEMG: 95.9±3.2%), FNC showed a percentage of task completion significantly lower (80.3±14.9%; *p*<0.005) with minimum values reaching 58%. The tracing error of subject S2 was more than two-fold higher using FNC (1.78±1.04 cm) than with FSC (0.54±0.34 m; *p*=0.002) or sEMG (0.71±0.35 cm; *p*=0.007). No significant differences were found for the smoothness metric, for which all methods presented mean values around -3 (Fig. 7, Table 5). While sEMG based control presented a performance similar to FSC in terms of tracing error, percentage of task completion and smoothness, the mean speed when using sEMG (2±0.3 cm/s) was significantly lower than with FSC (3.5±0.2 cm/s; *p*<0.001) or with FNC (3.4±0.7 cm/s; *p*<0.001). The results of the questionnaire showed a clear preference for sEMG with the exception of “being easy to set up”, for which subject S2 indicated that FNC was the easiest.

### Subject S3 - Brooke score: 6

Subject S3 could perform only one movement with FNC (shown in Fig. 6 but obviously not used for the data analysis), one repetition with FSC (N=5) and two repetitions with sEMG (N=10). The trajectories of subject S3 had a lower mean percentage of task completion and speed with FSC (PTC: 73.7±15.3%; SP: 1.5±0.04 cm/s) compared to sEMG (PTC: 85±7.1 %; SP: 2±0.6 cm/s). These differences were not statistically significant (Table 5), but note the low number of observations. The mean values of the tracing error and smoothness were similar between FSC and sEMG (Table 5). The results of the questionnaire showed a clear preference for sEMG, with the exception of accuracy and easy to control, for which subject S3 indicated that he preferred FSC. Additionally subject S3 indicated that FNC was the easiest to set up.

## Discussion

### Performance and Users’ acceptance

The results of the movement performance metrics and subjective preference of the three control methods differed with the level of arm function (i.e. Brooke score) of the participants. All subjects were asked to perform the same number of repetitions per control method, yet in some cases the participants were not able to complete the full number of tasks. We consider that the fact that subjects could not perform a task due to fatigue or lack of force is a relevant outcome of the study that reveals the limitations of some control methods. Subject S1 (Brooke 4) showed a high overall performance completing all three repetitions with all three control methods, and presents the best movement performance when using the FNC method. The low number of repetitions, the low percentage of task completion, and the high tracing error of FNC clearly indicated that subject S2 (Brooke 5) performed better with FSC and sEMG than with FNC. Even though the movements of subject S2 presented significantly lower speed with sEMG than with FSC, he preferred sEMG over FSC since the latter was perceived to be fatiguing. Finally, subject S3 (Brooke 6) was not able to effectively use neither of the force-based control interfaces (i.e. no movements completed with FNC and one repetition completed with FSC) and showed a better performance in terms of percentage of task completion and speed with sEMG and could reach almost his entire workspace. The results of the questionnaire indicated that S2 and S3 had a clear preference for sEMG, and subject S1 preferred FNC. The subjective preferences of the participants were in accordance with the results of the performance metrics.

### Participants and experimental protocol

Any study dealing with testing assistive devices for subjects with DMD is severely conditioned by the low density of available candidates. In our case, the limitation of subjects was also a consequence of our commitment to cause the least inconveniences to the subjects, and strictly observing the legal and ethical constraint that restricts the participation of any given subject to one single study at the same time.

Our conclusions need to be regarded with caution due to the low sample size. A higher number of participants would have resulted on stronger conclusions, but the specificities of adults with DMD do not make purely academically-oriented studies with a high number of participants advisable. Once the current exploratory phase will be completed, stronger conclusions should rather come as an added value from tests for fitting personalized assistive devices to be used in real life, provided these tests are conveniently designed with standardized protocols.

Adults with DMD experience strong training and fatigue effects due to the disuse of their arms. Therefore, the present experimental protocol was designed to balance the amount of training and evaluation trials: the protocol allowed the participants to learn how to use the control interfaces and perform the experimental task, while having a sufficient number of evaluation trials per condition, but keeping the overall length of the experiment within five hours (including breaks) to minimize mental and physical fatigue. Reaching this balance was challenging and future studies must keep paying particular attention to the design of suitable protocols. Moreover, we consider that due to the small number of participants included in this study and the high functional variability between them, randomizing the order of the control methods would not have been effective.

### Passive vs. active support

As we already mentioned in the *Background* section, the FNC method resembles the dynamics of passive planar arm supports with a certain mass (*M*_*vir*_) and damping (*D*_*vir*_). FNC was only usable for subject S1 (Brooke 4), FSC was usable for subject S1 and S2 (Brooke 5), and sEMG control was usable for subjects S1, S2 and S3 (Brooke 6). These results suggest that men with a voluntary force above the stiffness forces can benefit from passive planar arm supports (i.e. FNC), and when voluntary forces decrease below the intrinsic force of the arm the effective use of passive arm supports is considerably reduced as these will hardly react to their intention (see maximum planar forces in Table 1). Thus, weaker subjects can benefit more from active arm supports that either actively compensate stiffness force, or are sEMG-controlled.

### Force vs. sEMG-based control

The use of force-based control interfaces for people with severe muscular weakness requires the distinction of the voluntary force of the user from the intrinsic force of the arm, or external disturbances from the environment. In this study the stiffness forces were estimated and actively compensated using a measurement-based method that created a two-dimensional force field (see Fig. 6). Our results showed an increase in the functional ROM of subject S2 and S3 when using FSC compared to FNC (S2: 409 vs. 557 cm^2^; S3: 132 vs. 248 cm^2^). Subjects S2 and S3 showed a clear benefit in terms of ROM since their maximum forces (S1: 20N; S2: 6N; S3: 3N) were equal or below the stiffness forces (see Fig. 6). However, both subjects also reported that FSC was too fatiguing for them, as we also found in [24]. Differently, for subject S1, the active compensation of the stiffness forces made the control of his movements less stable and smooth than with FNC, which forced him to reduce the movement speed to keep a low tracing error. Additionally, subject S1 also reported a lower effort/fatigue when using FNC, which suggests that probably he had to actively increase his joint impedance (and effort) when using FSC.

The sEMG signals are not affected by the intrinsic forces of the arm (Fig. 3) and therefore we presume that they can better produce the intended movement of people with voluntary forces below the intrinsic forces of the arm. While we found that functional ROM of all subjects when using sEMG was similar to or larger than when using FSC, sEMG was reported by subject S1 and S3 as less intuitive and required longer training than force-based control. Additionally, sEMG-based control presented a significantly lower movement speed compared to force-based control methods in subject S1 and S2. However, it is worth noting that the differences in speed between sEMG- and force-based control are probably caused by the difference in the admittance parameters.

In the context of assistive devices for people with severe muscular weakness such as DMD, we consider that the usability of the device is highly affected by the fatigue of the user. The results of this study and the ones from [24] suggests that sEMG-based control allows the performance of one- and two-DOF arm movements with lower levels of fatigue compared to force-based control. Special consideration must be given to this observation as it suggest that sEMG could be a better solution for adults with DMD, although objective and quantitative studies on fatigability should be undergone for a more sound choice. In any case, the final decision must be guided by the specificities of the patient: for example, our results also suggest that force-based control interfaces with active gravity and stiffness compensation can be a better alternative for those cases in which voluntary forces are higher than the intrinsic forces of the arms.

### Implementation

The assistance of planar arm movements can enable the performance of tabletop tasks such as computer/phone/table use, writing or drawing, or the control of the wheelchair’s joystick. We have recently developed a two-DOF active arm support known as the A-Arm [30], which assists movement in the horizontal plane and replaces the normal arm rest of a wheelchair. In the A-Arm we have implemented the same control interfaces as the ones evaluated in this study. A preliminary pilot evaluation with one adult with DMD (24 years-old, Brooke 5) indicated that the assistance provided by the A-Arm enabled him to move the arm in the horizontal plane and perform task like reaching (and using) his mobile phone, laptop or wheelchair joystick – actions that he could not do without the A-Arm. Being able to perform this planar movements was perceived by the subject as a significant increase of autonomy.

### Extension to three-DOF control

While we have seen that the control of two DOF can support the performance of some basic ADL, the extension to three controllable DOF would increase the support capabilities of the assistive device. Extending the control interfaces to operate three-DOF presents challenges for both sEMG and force-based control methods. In the case of force-based control, the measurement of gravity and stiffness forces would need to be expanded to three-dimensions, which would result in a long measuring session probably hindering users’ acceptance. An alternative approach would be the modeling of the gravity and stiffness forces as proposed in [36] to reduce the duration of the measuring session. In any case, force-based control interfaces will always be compromised by the challenging detection of the low-amplitude voluntary forces of people with severe muscular weakness.

The sEMG-based control method implemented in this study is based on the clinical standard sEMG-based control strategy commonly used in upper limb prosthetics and known as direct or proportional control. The direct sEMG-based control method is robust but requires the user to generate independent sEMG signals, which can be mentally and physically fatiguing, and provide a limited number of simultaneously controlled DOF [13, 34]. In a previous pilot study [29], we tried to extend the sEMG-based control presented in this paper with a third DOF that controlled the height of the end-point. To this end, we used two additional electrodes that were located on the middle part of the deltoid muscle and on the lattisimus dorsi muscle. We found that subjects were not able to simultaneously control all three DOF, and therefore we included an extra electrode to function as a switch between the control of horizontal and vertical DOF. Future research will focus on further evaluating the feasibility of three DOF control with and without the switching function.

In upper extremity prosthetic applications, more advanced sEMG-based control methods such as regression-based algorithms have shown simultaneous and proportional control over two DOF with sEMG signals [37] and three DOF with intramuscular EMG signals [29]. These methods do not require isolated EMG signals as they can learn to map complex activation patterns to specific movements, and provide proportional and simultaneous control of several DOF. However, these methods usually required a larger number of electrodes and long preparation and calibration times compared to direct sEMG control. We consider that for a control interface that needs to be used in daily life, low preparation time and low number of (re)calibrations, are important requirements. Future research will also include developing robust sEMG regression-based algorithms that can give people with DMD control over three DOF.

In the present study we measured sEMG signals from muscles that are not directly related to the supported movement by controlling the arm in task-space. Even though it has been shown that humans can quickly adapt to non-intuitive mappings of sEMG to movements [38], the use of an exoskeleton as arm support could improve the level of control intuitiveness by mapping the sEMG signals to joint torques/movements.

## Conclusions

We were able to evaluate the feasibility, performance and users’ preference of sEMG-based control, force-based control with stiffness compensation (FSC) and force-based control with no compensation (FNC) during planar line-tracing tasks in three adults with DMD. Movement performance and subjective preference of the three control methods differed with the level of arm function (i.e. Brooke score) of the participants. Our results indicate that all three control methods have to be considered in real applications and future studies, as they present complementary advantages and disadvantages. The fact that two of the subjects experienced the sEMG-based control interface as less fatiguing than the force-based control interfaces (also found in our previous study [24] involving elbow movements) must be given special consideration and suggests that sEMG-based control interfaces could be a better solution for adults with DMD, although objective and quantitative studies on fatigue effects should be undergone for a more sounded choice. In any case, the final decision must be guided by the specificities of the user: for example, our results also suggests that force-based control interfaces (with active gravity and stiffness compensation) can be a better alternative for those cases in which voluntary forces are higher than the intrinsic forces of the arms.

## Endnotes

^1^ This study builds upon the work presented at the 14th edition of the IEEE/RAS-EMBS International Conference on Rehabilitation Robotics (ICORR) in 2015, Singapore [29, 39].

^2^ Bold font style symbols indicate vectors and regular font style symbols indicate scalars.

## Abbreviations

ADL: Activities of daily living
DMD: Duchenne muscular dystrophy
DOF: Degree(s) of freedom
FNC: Force no compensation
FSC: Force stiffness compensation
PTC: Percentage of task completion
ROM: Range of motion
sEMG: Surface electromyography
SPARC: Spectral arc length
SM: Smoothness
SP: Speed
TE: Tracing error
